# Arthroscopic treatment of osteoid osteoma in the posterior proximal tibia: A case report and literature review

**DOI:** 10.1097/MD.0000000000037076

**Published:** 2023-02-02

**Authors:** Yinhao He, Xiaosheng Li, Zhi-Xing Tu, Hong-Wen Chen, Hui Zeng, Qiang Peng, Tie-Zhu Chen

**Affiliations:** a Hunan Provincial People’s Hospital (The First Affiliated Hospital of Hunan Normal University), Changsha, Hunan Province, China; b Fujian Medical University, Fuzhou, Fujian Province, China.

**Keywords:** arthroscopy, case report, knee joint, osteoid osteoma, posterior tibia

## Abstract

**Background:**

Osteoid osteoma (OO) is a benign lesion characterized by an increased fibrous component in the bone marrow, presence of bone-like structures within the medullary cavity, and a surrounding sclerotic bone rim. Reports on OO located in the posterior proximal tibia are rare.

**Case summary:**

Herein, we report the case of an 18-year-old male, admitted for the evaluation of right knee pain. The right knee pain had started 6 months prior without any apparent cause, which was notably severe at night, affecting sleep, and was exacerbated while climbing stairs or bearing weight. The patient also experienced pain on flexion. Three-dimensional computed tomography and magnetic resonance imaging revealed a nodular lesion beneath the cortical bone of the posterior medial plateau of the right tibia and an abnormal signal focus on the posterior lateral aspect of the right tibial plateau associated with extensive bone marrow edema. A small amount of fluid was present in the right knee joint capsule. The patient subsequently underwent arthroscopic excision of the OO. Postoperatively, there was significant relief of pain, and the knee range of motion returned to normal.

**Conclusion:**

Although OO in the posterior proximal tibia is a rare occurrence, it can be effectively excised through minimally invasive arthroscopic visualization.

## 1. Introduction

Osteoid osteoma (OO) is a common benign bone tumor in orthopedics, characterized by a central core of vascular bone-like tissue surrounded by a sclerotic bone rim, often referred to as the “nidus sign.”^[1]^ It was first described by Bergstrand^[2]^ in 1930 and further adopted and defined by Jaffe in 1935.^[3]^ OO predominantly affects individuals at the age of 5 to 25 years,^[4]^ with incidence rates of 13% in those aged > 30 years and 3% in children aged < 5 years. It exhibits a male-to-female ratio of 3:1.^[5,6]^ OO accounts for approximately 10% to 14% of all benign bone tumors and 2% to 3% of all primary bone tumors.^[5,7]^ Another characteristic of OO is its size, typically not exceeding 2 cm. It primarily occurs in long tubular bones, with the femur and tibia being the most common sites.^[4]^ Interestingly, 60% OOs are located near the hip joint or in the femur midshaft, rather than the knee joint.^[8]^ Consequently, reports on arthroscopic excision of OO in the posterior proximal tibia are rare.

Herein, we report a case of OO located in the posterior proximal tibia of a young male who underwent arthroscopic excision of the lesion.

## 2. Case presentation

### 2.1. Chief complaint

An 18-year-old male patient was admitted to the hospital with right knee pain for 6 months.

### 2.2. Present medical history

The patient experienced right knee pain 6 months prior without any apparent cause. The pain was intermittent, dull, and aching. It significantly affected his sleep and exacerbated while climbing stairs or bearing weight, whereas bed rest provided some relief. The pain was not significantly influenced by seasons or weather conditions and was not associated with swelling or restricted movement. The patient experienced pain on deep flexion without any locking or catching sensation. During periods of pain relief, normal activities were possible. He sought admission to our department for further treatment. He did not experience chills or fever and reported normal appetite, sleep, and regular bowel and bladder habits. There was no significant weight loss observed.

### 2.3. Past medical history

There was no history of infectious diseases such as hepatitis, tuberculosis, or malaria; hypertension; heart disease; recent aspirin intake; diabetes; cerebrovascular disease; psychiatric disorders; surgeries; traumas; or blood transfusions. He had no known allergies to substances, medications, or foods. The patient vaccination history was not available.

### 2.4. Personal and family history

The patient had no significant personal or family medical history. There were no known cases of hypertension, diabetes, hepatitis, pulmonary tuberculosis, or allergies to medications or foods in the family.

### 2.5. Physical examination

Both the knee joints exhibited no deformities. Tenderness was noted in the posterior lateral gap of the right knee joint. Skin temperature was normal. Floating patella sign was positive, patellar grind and lateral stress tests and Murray sign were negative, deep flexion and hyperextension tests were partially positive. Both the knee joints showed negative results for anterior and posterior drawer tests and Lachman sign. Range of motion for both knee joints was 0° to 110°. Longitudinal and axial percussion tendernesses were absent. Muscle strength in both the lower limbs was graded as V. Sensation in both the lower limbs was symmetrical. Dorsalis pedis pulses were bilaterally palpable.

### 2.6. Laboratory examinations

Preoperative white blood cell and lymphocyte counts and percentage of neutrophils were all elevated.

### 2.7. Imaging examinations

**Figure 1.**
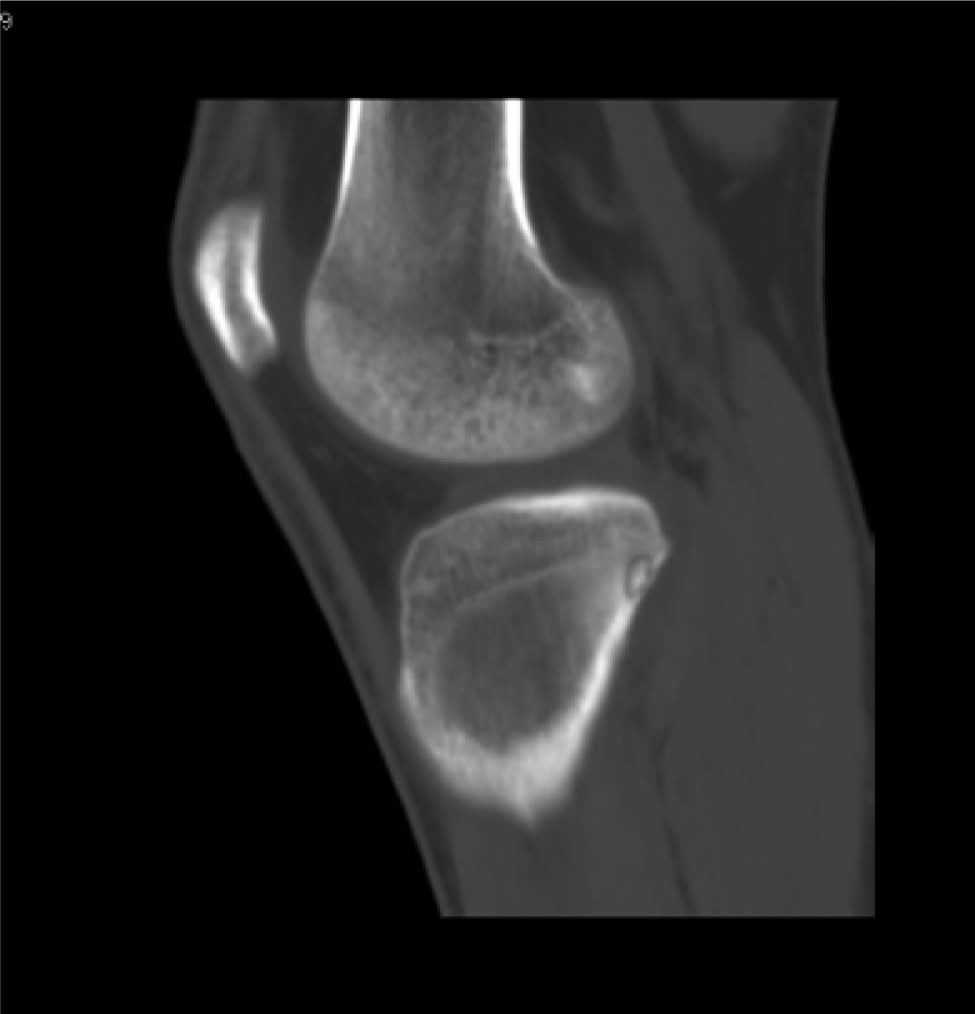
Preoperative CT image in mid-sagittal plane. CT = computed tomography.

**Figure 2.**
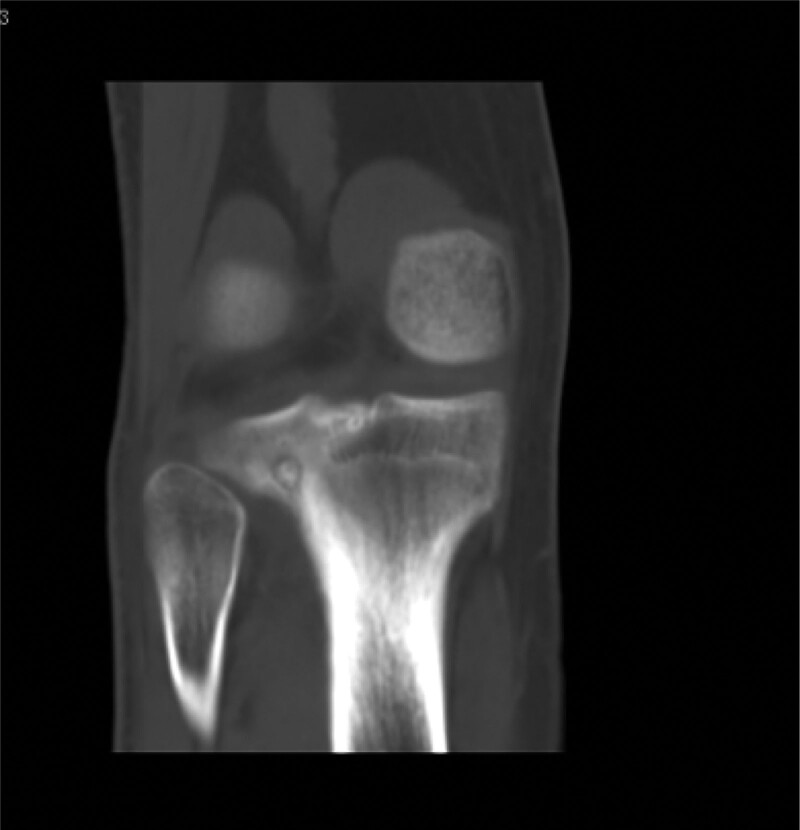
Preoperative CT image in coronal plane. CT = computed tomography.

**Figure 3.**
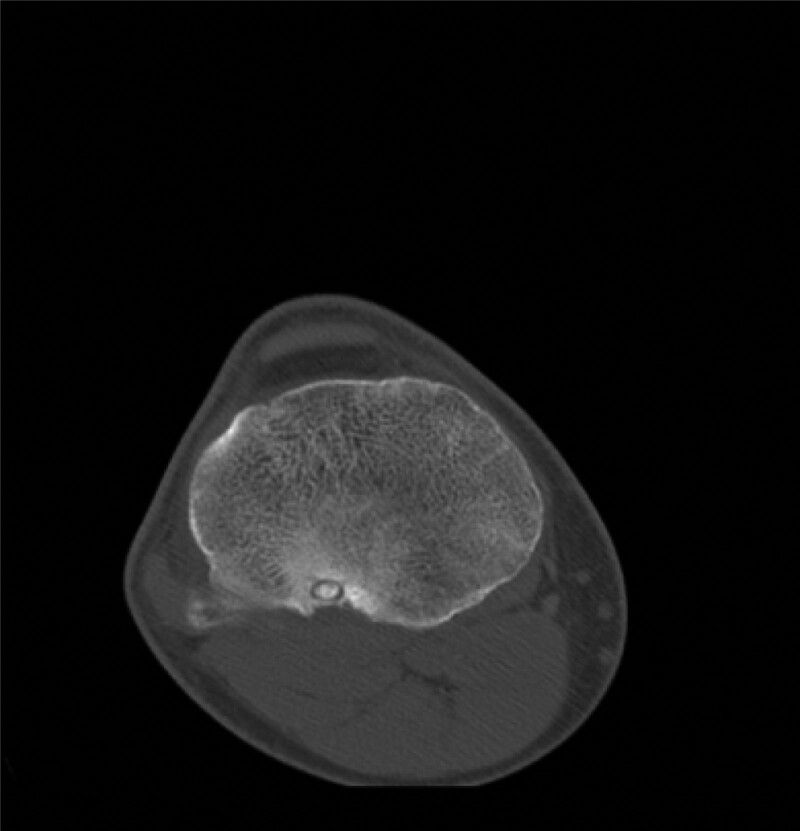
Preoperative CT image in transverse plane. CT = computed tomography.

**Figure 4.**
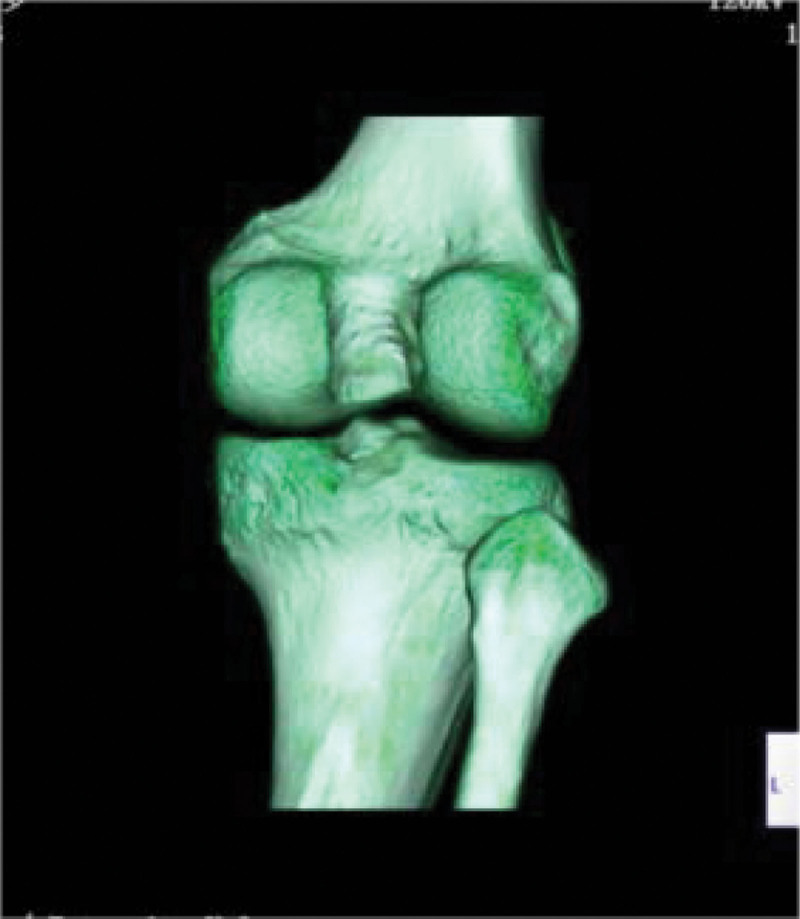
Preoperative three-dimensional CT image. CT = computed tomography.

Computed tomography (CT) (Figs. 1–4) showed a nodular lesion beneath the cortical bone of the posterior medial plateau of the right tibia, nature of which was undetermined (OO or other pathologies). There was a small amount of effusion in the right knee joint capsule.

**Figure 5.**
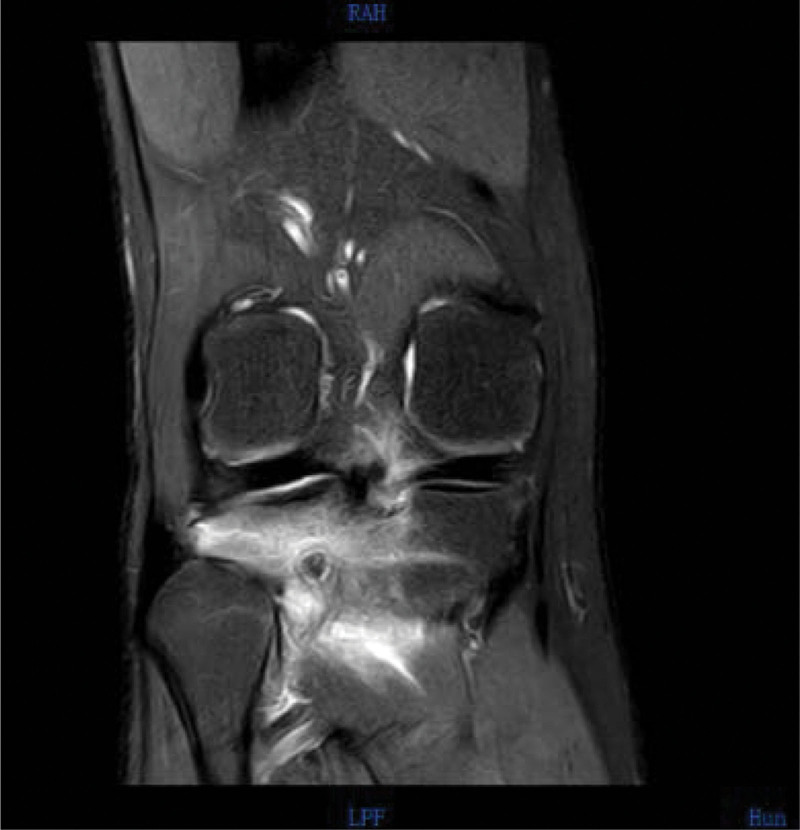
Preoperative MRI in coronal plane. MRI = magnetic resonance imaging.

**Figure 6.**
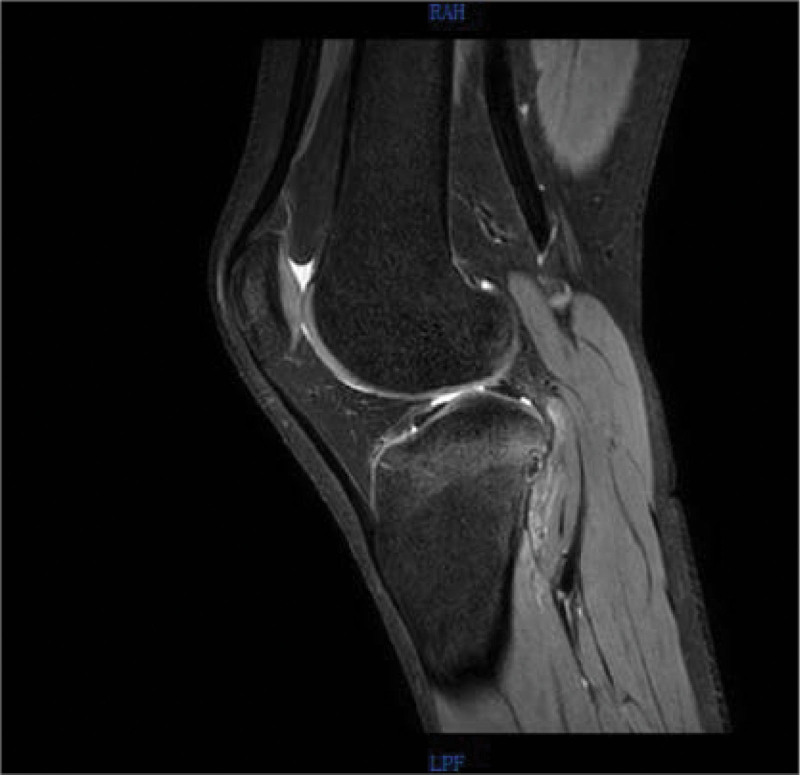
Preoperative MRI in mid-sagittal plane. MRI = magnetic resonance imaging.

**Figure 7.**
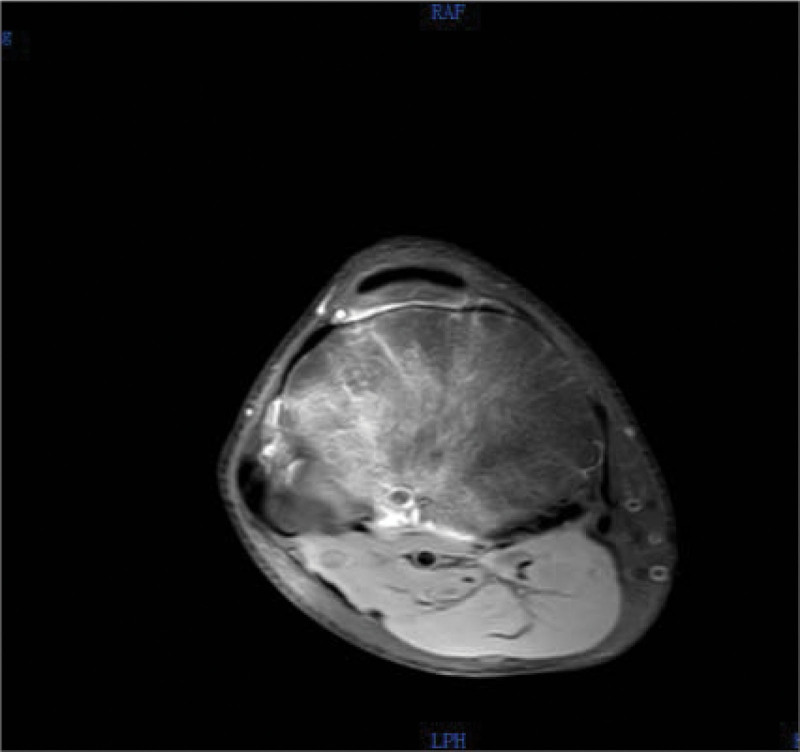
Preoperative MRI in transverse plane. MRI = magnetic resonance imaging.

**Figure 8.**
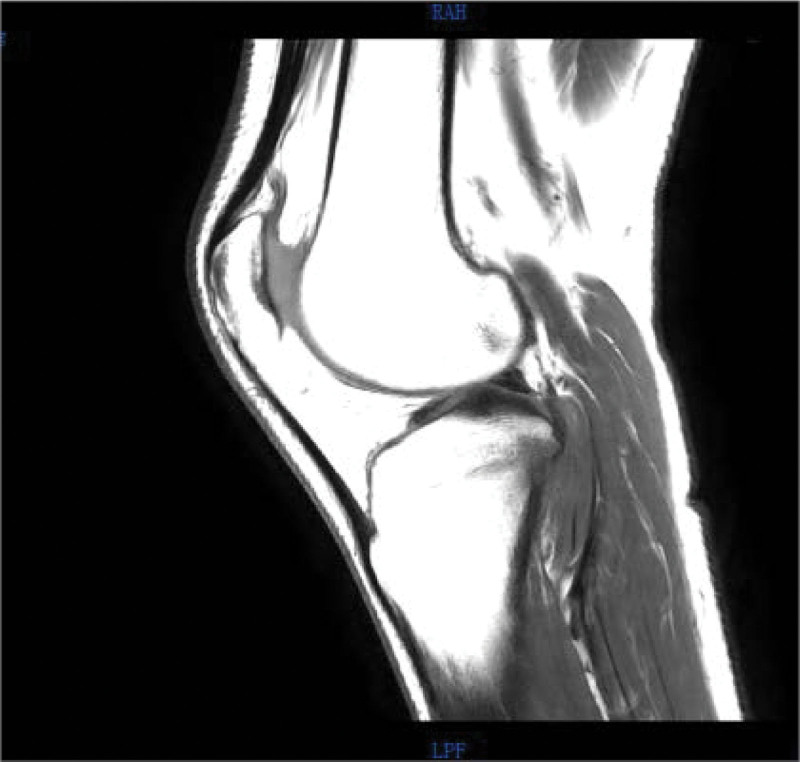
Preoperative MRI T1 sequence in sagittal plane. MRI = magnetic resonance imaging.

Magnetic resonance imaging (MRI) (Figs. 5–8) showed abnormal signal focus with extensive bone marrow edema on the posterior lateral aspect of the right tibia, possibly indicative of an OO. Additionally, there was a small amount of effusion in the right patellar bursa and joint cavity.

## 3. Final diagnosis

Pathological examination of the chondral and fibrous tissues in the right tibial lesion revealed OO, consistent with the clinical and radiological presentation. The final diagnosis was OO.

## 4. Treatment

**Figure 9.**
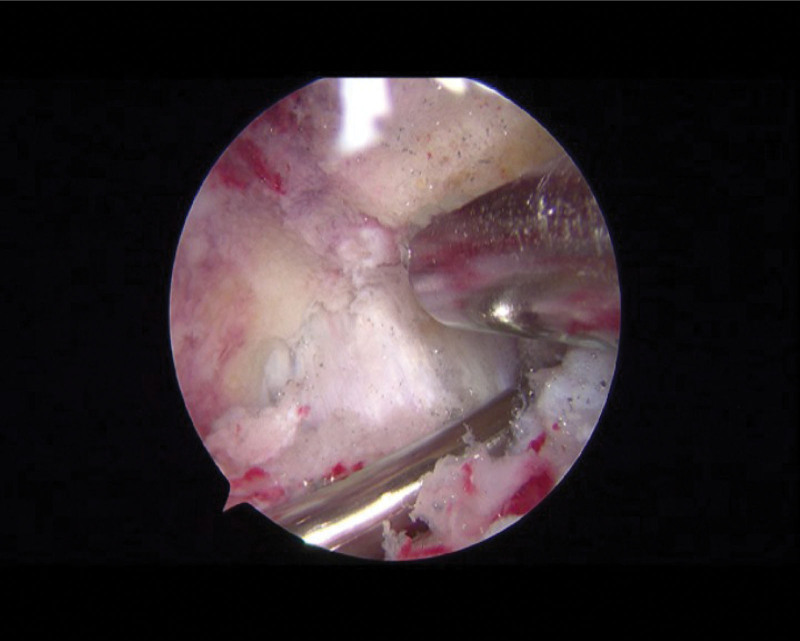
Intraoperative arthroscopic examination; intraoperative arthroscopic debridement of the OO lesion. OO = osteoid osteoma.

**Figure 10.**
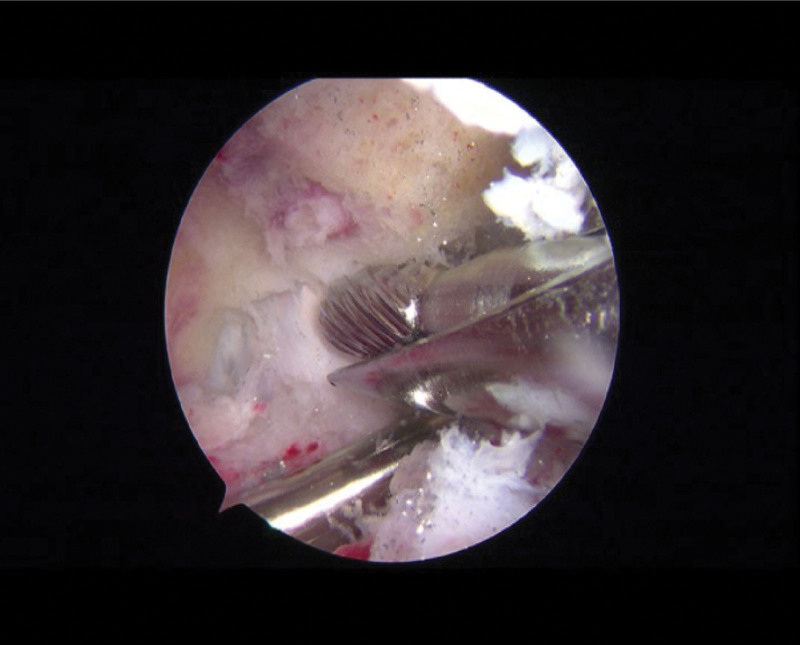
Intraoperative arthroscopic examination; intraoperative arthroscopic debridement of the OO lesion. OO = osteoid osteoma.

The surgical procedure was as followed: The patient was placed in a supine position on the operating table, and a tourniquet was applied at the root of the thigh. After general anesthesia induction and routine disinfection, a mixture of 60 mL 0.9% saline solution and 0.1 mg adrenaline was injected into the joint cavity. Incisions of approximately 0.5 cm were made on both sides of the patellar ligament, 1 cm below the lower patellar edge, serving as the portal and instrument entry points. Arthroscopic exploration (Fig. 9) was performed in a sequential manner as previously described.^[9,10]^ After identifying the lesion area of the OO, a posterior medial approach was established. The posterior intermeniscal ligament and posterolateral compartment were exposed by opening the posterior longitudinal septum. A posterior lateral approach was established, and the synovial congestion was cleared posterior and inferior to the lateral meniscus. Localized reddening of the bone was observed upon inspection, and the abnormal tissues were thoroughly cleared using drilling until normal bone was reached (Fig. 10). Curettage and laser ablation were performed to remove synovial hyperplasia, followed by radiofrequency ablation of synovial congestion and edema. The damaged area of the cartilage surface was trimmed. Upon re-inspection of the joint cavity, no actively bleeding points were observed. The incisions were closed, and the joint cavity was injected with tranexamic acid, ropivacaine, and sodium hyaluronate. Elastic bandage was applied. The surgery proceeded smoothly, and the excised tumor was sent for pathological biopsy. The surgery was successful, and the anesthetic effect was satisfactory. There was minimal intraoperative bleeding, and no blood transfusion was required.

## 5. Outcome and follow-up

### 5.1. Postoperative pathological results

**Figure 11.**
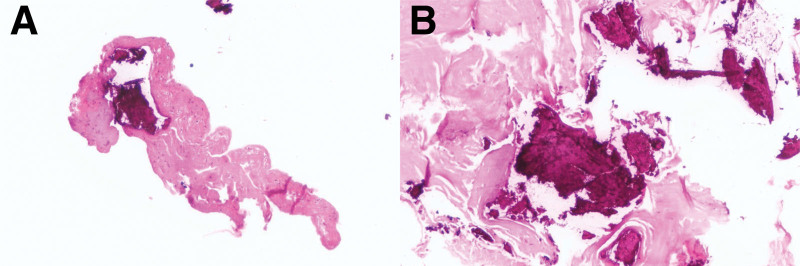
(A) Pathological biopsy results: visible osteoid bone tissue (hematoxylin-eosin staining × 100). (B) Pathological biopsy results: visible osteoid bone tissue (hematoxylin-eosin staining × 100).

**Figure 12.**
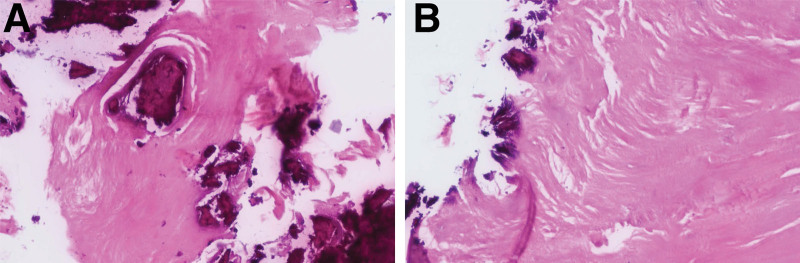
(A) Pathological biopsy results: visible osteoid bone tissue (hematoxylin-eosin staining × 200). (B) Pathological biopsy results: visible osteoid bone tissue (hematoxylin-eosin staining × 200).

Pathological biopsy reports (Figs. 11 and 12) confirmed the presence of OO within the lesion located in the posterior aspect of the tibia. This diagnosis aligned with the clinical history, symptoms, and radiological findings, thus establishing the definitive diagnosis of OO.

### 5.2. Follow-up imaging results

**Figure 13.**
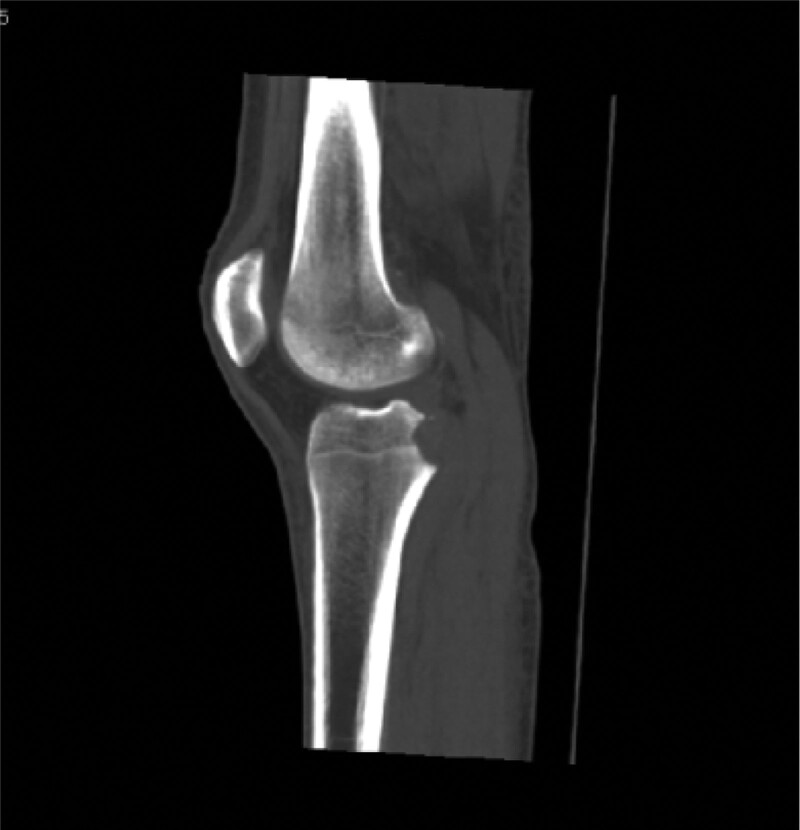
Postoperative CT image in mid-sagittal plane. CT = computed tomography.

**Figure 14.**
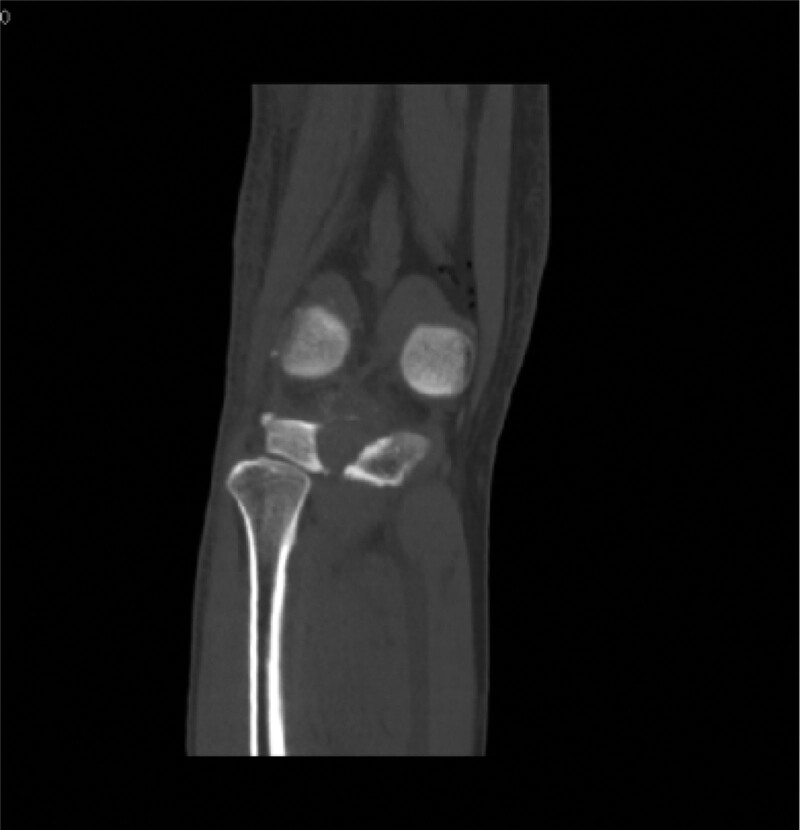
Postoperative CT image in coronal plane. CT = computed tomography.

**Figure 15.**
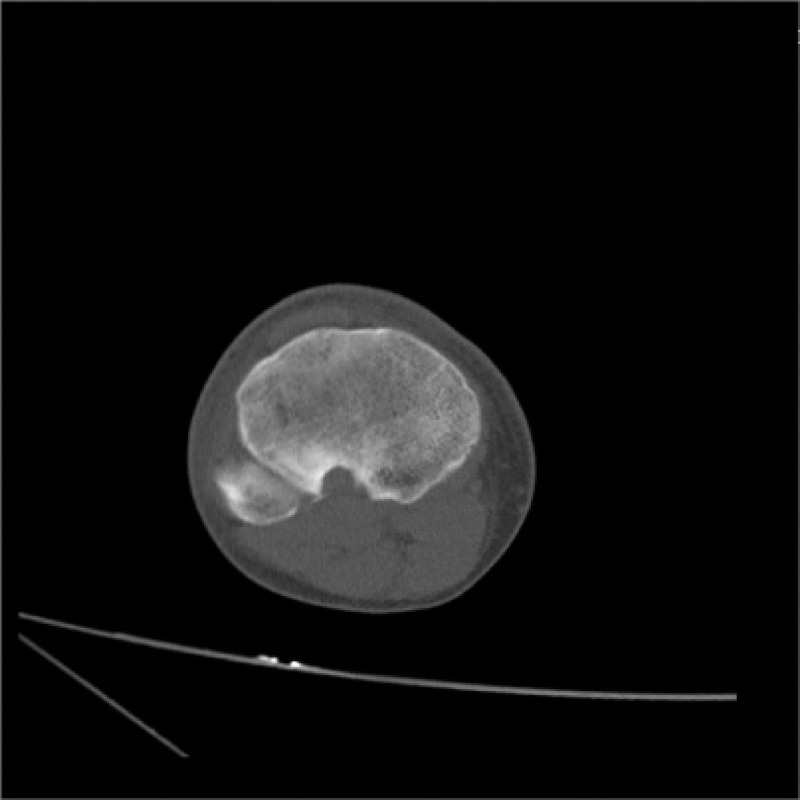
Postoperative CT image in transverse plane. CT = computed tomography.

**Figure 16.**
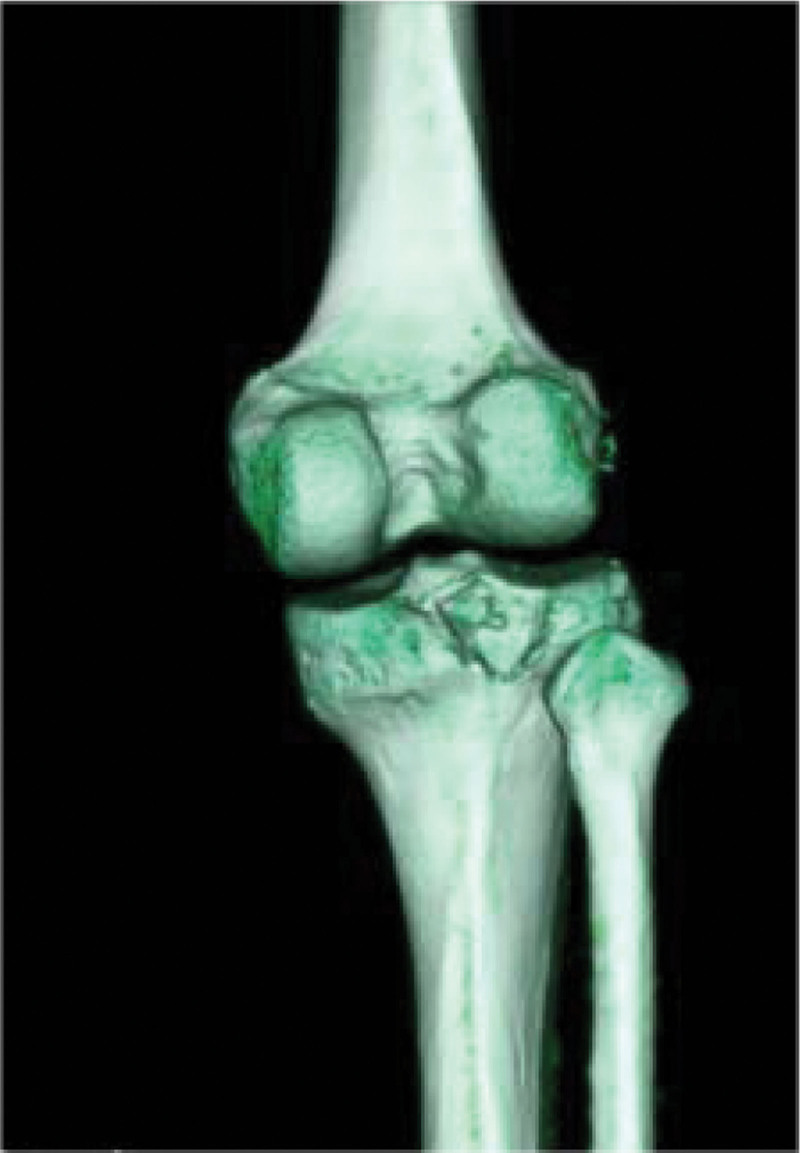
Postoperative three-dimensional CT image. CT = computed tomography.

Figures 13–16 shows the postoperative CT findings. Three-dimensional (3D) CT image following arthroscopic examination of the right knee revealed cleaned and trimmed articular cartilage lesions and resolution of joint contractures. Postoperative CT showed a reduction in intra-articular gas accumulation compared with the earlier images.

The patient leg was wrapped in a cotton bandage with an adjustable knee joint support for immobilization. Within 24 hours after surgery, the patient experienced significant pain relief and reported a noticeable improvement in the quality of nighttime sleep. On the second day, the patient was able to ambulate with the aid of crutches, with the affected limb not bearing weight. Within 4 weeks, the knee range of motion reached 120°. At 3 months postoperatively, the external immobilization support was removed, allowing for normal daily activities. By 6 months, the patient was able to jog. At 1 year, the joint exhibited normal flexion-extension movement, enabling participation in regular sports activities.

## 6. Discussion

### 6.1. Diagnosis

In 1966, Edeiken^[11]^ classified OO into cortical, cancellous, and subperiosteal types. OO often manifests as a solitary cortical lesion in a skeletal segment.^[7]^ In rare cases, multiple centers or lesions may occur within the same skeletal area.^[12]^ Tibial OO is a benign lesion characterized by increased marrow fibrous components and the presence of osseous structures within the marrow cavity.

Clinical symptoms and morphology of intra-articular OO are atypical. Extra-articular tibial OO often presents with nighttime pain, swelling, and restricted mobility. Nighttime pain is the predominant clinical symptom of extra-articular OO,^[13]^ reaching up to 80% in some cases.^[14]^ The pain mechanism remains unclear. Kawaguchi^[15]^ suggested that prostaglandin products generated within tumor tissues may lead to changes in vascular pressure, stimulating local nerve endings. This viewpoint is primarily supported by the presence of non-myelinated nerve fibers.

Currently, the consensus is to use radiography, MRI, radiographic nuclear bone scanning, and 3D CT examination for the diagnosis of knee joint OO. Radiographic features of OO include a lesion surrounded by hardened edges that can be penetrated by X-rays. Therefore, extra-articular OO is difficult to diagnose with radiography (in 78% of cases),^[16]^ and is prone to misdiagnosis as osteosarcoma.^[17]^ Currently, 3D CT examination is considered the gold standard for noninvasive diagnosis of tibial OO.^[17]^ MRI provides a better evaluation of joint structures, allowing for improved differentiation between intra- and extra-articular OO and other tumor lesions, while preserving nearby neurovascular structures related to OO.^[18]^ However, due to the high sensitivity of MRI in detecting joint effusion, bone marrow edema, and synovitis,^[13]^ it often tends to mask the condition and can lead to misdiagnoses such as patellar chondromalacia and peeling osteochondritis,^[1]^ with a misdiagnosis risk as high as 35%.^[5]^ Radionuclide bone scanning has high sensitivity in diagnosing OO, with the double-ring sign being a typical manifestation. However, due to the acute phase of OO and synovial hyperplasia and congestion, there may be imaging inaccuracies.^[2]^ Pathological examination is often the diagnostic criterion for OO, but some doctors believe that it is unnecessary to perform a pathological biopsy, which can lead to misdiagnosis as osteosarcoma.^[19]^ Therefore, based on the patient condition, combined with clinical symptoms, imaging, and pathological reports, the diagnostic rate of the disease can be improved, and personalized diagnosis and treatment plans can be formulated to reduce patient suffering, lower misdiagnosis rates, and perioperative diagnostic and treatment costs.

### 6.2. Treatment

#### 6.2.1. Limitations and challenges of traditional treatment of tibial OO.

Conservative treatment with oral salicylates is often the treatment choice when pain is significant. Salicylates can effectively control pain. When conservative treatment is ineffective or when conditions worsen, such as deformity, neurological symptoms, and tumor growth, further treatment should be considered.

The principle of surgical treatment is the accurate and complete removal of the nest of the OO and surrounding reactive sclerotic bone. In 1935, Jaffe^[3]^ performed the first complete excision of OO. For a long time, traditional open-excision surgery carried the risks of prolonged hospitalization and potential recurrence due to inadequate removal of the lesion. Open-excision surgery can result in large bone defects. To avoid postoperative fractures, further bone grafting and internal fixation are required.^[20]^ The internal fixator should be removed after 1 year, which can cause secondary damage and affect the growth and development of minors. With the development of minimally invasive techniques, when the lesion is often solitary, percutaneous radiofrequency ablation under CT guidance is the preferred treatment for extra-articular OO, with a success rate of up to 95%.^[6]^ However, joint OO often attaches to the cartilage and nearby tissues. Radiofrequency may lead to cartilage injury and soft tissue contusion, and in severe cases, exacerbate degenerative changes in the joint cartilage.^[21]^ Additionally, there are certain limitations in collecting pathological samples during operation^[22]^; at the same time, percutaneous radiofrequency ablation under CT guidance may lead to risks such as skin necrosis, nerve paralysis, osteomyelitis, and fractures.^[8]^

#### 6.2.2. Development and application of arthroscopic treatment for tibial OO.

In 1986, Heuijerjans^[19]^ first reported successful arthroscopic excision of tibial OO. In recent years, with the arthroscopic visualization of the complete excision of intra-articular lesions, exploration of the joint cavity and surrounding interval tissues, small postoperative incisions, rapid incision recovery, short hospital stays, satisfactory success rates, and improved prognosis have all been achieved. This has been widely promoted in the field of orthopedics.^[23,24]^

Alrassasi et al^[25]^ conducted a case analysis of a 28-year-old female patient with OO of the right elbow. The patient had contracture of the right elbow, with a range of motion between 50° and 110°. Arthroscopic excision of the OO, synovectomy, and capsular release were performed. One year after surgery, all pain symptoms completely disappeared, and the right elbow range of motion increased to 30° to 145°. Knezevic et al^[23]^ conducted a case-control analysis of patients with elbow joint OO, treated with arthroscopic radiofrequency ablation and percutaneous radiofrequency ablation under CT guidance by the same surgeon at the same institution over approximately 6 years. They stated that arthroscopy can precisely locate the lesion before radiofrequency ablation, collect samples for pathological biopsy, further confirm the tumor diagnosis, and minimize damage to the cartilage and surrounding soft tissues during the surgery. In contrast, percutaneous radiofrequency ablation under CT guidance can only clear the lesion, making it difficult to make a definitive diagnosis. Ge et al^[26]^ summarized recent studies on arthroscopic treatment of upper limb joint OO. Nineteen high-quality studies were selected for retrieval and analysis, including 19 cases of shoulder joint OO, 93 cases of elbow joint OO, and 8 cases of wrist joint OO. They concluded that arthroscopic treatment of upper limb OO is successful, without tumor recurrence or complications. However, due to the narrow location of some joint lesions, the arthroscopic view is poor, which poses a risk of incomplete excision. Spiker et al^[27]^ conducted a retrospective analysis of clinical data from 33 patients with hip joint OO. The patients underwent hip arthroscopic excision, which removed the OO lesions and relieved impingement symptoms. After surgery, the scores of hip joint function, including Hip Outcome Score-Activity of Daily Living, modified Harris Score, and International Hip Outcome Tool, significantly improved. Postoperative pain symptoms were significantly relieved. Rolvien^[16]^ conducted a retrospective study on 367 patients with joint OO who underwent knee arthroscopic excision at the same medical center over a period of 42 years. Ninety-seven of them had extra-articular tibial OO, confirmed by pathological biopsy. Three months after follow-up, the patients had no obvious pain, and appropriate exercise could be performed. Park^[20]^ used a 3D printed navigation- positioning device to guide the arthroscopic excision of intramedullary OO in the posterior tibia. The incision was small, operation time was short, and postoperative recovery was rapid. Three months after surgery, the patient resumed physical activity, and the functional scores were excellent (Lysholm score 94, WOMAC score 0). Plecko et al^[8]^ performed knee arthroscopic excision of OO in 4 cases of intra-articular OO between 2005 and 2015. The average follow-up period was 24 months. After surgery, pain symptoms were significantly relieved, and there were no other abnormalities.

The above studies show that all patients successfully underwent arthroscopic excision surgery for OO. Compared with the preoperative state, postoperative pain was effectively relieved, and visual analogue scale scores significantly improved. At the same time, the joint function of the patients improved significantly. In addition, there were no severe complications after surgery. Only a few patients experienced mild symptoms such as infection, bleeding, and nerve damage, which were actively treated and recovered after symptomatic support. Arthroscopic excision of OO is effective in various fields of orthopedics. The removal of the “nest” of the tumor core and the surrounding sclerotic bone can eliminate pain and cure the patient. In this study, arthroscopic minimally invasive visualization was performed around the patient knee joint through a small incision established in the posterior knee joint. This approach avoided extensive incision of the skin and soft tissues. Additionally, most OO lesions were excised and sent for pathological biopsy using an osteotome. The remaining lesions were then thoroughly cleaned in a meticulous manner to avoid excessive bone defects. After active postoperative rehabilitation exercises, pain was significantly relieved, sleep normalized, knee joint movement resumed, and normal walking and living were resumed after 6 months. However, it is worth considering that doctors should comprehensively consider factors such as the patient age, patient demands, lesion location, severity of the lesion, and perioperative risk when choosing a treatment method, and weigh the pros and cons to formulate an individualized diagnosis and treatment plan. It can be concluded that arthroscopic treatment of tibial OO is a safe and effective treatment method. However, from the anatomical analysis of the knee joint, the knee joint is the most complex joint in the human body with rich blood vessels and nerves in the posterior part. Therefore, senior orthopedic surgeons need to operate carefully to reduce damage to the blood vessels, nerves, and surrounding soft tissues. Currently, the literature on arthroscopic treatment of posterior tibial OO is limited, and more clinical research and analysis are needed to further verify the effectiveness and safety of this treatment method.

### 6.3. Limitations

Only a few case reports were available for the literature review, which might have led to selection bias.^[28]^ In some narrow joints, there is a risk of unclear arthroscopic vision and incomplete excision.^[26]^ At the same time, there is a long clinical learning curve for arthroscopy, and there may be differences between different surgeons, leading to the possibility of misdiagnosis and incomplete excision during surgery.

## 7. Conclusion

Through case reports, we have found that although OO in the posterior tibia is rare, it can be removed under knee arthroscopy with minimally invasive visualization. This approach offers advantages such as small incisions, short operation times, low infection risks, effective removal of tibial lesions, significant relief of pain and other discomforts, restoration of damaged tissues, promotion of patient recovery, shortened hospital stays, no recurrence, maximal preservation of normal tissues, sufficient specimens for pathological examination, and rapid postoperative recovery. This indicates that arthroscopic treatment of tibial OO is a safe and effective option.

**Table 1:** Cases of OO excised at different sites in the literature.

Authors	Number of cases	Yr	Gender	Average age (yr)	Location of lesion	Diagnostic imaging	Treatment	Average follow-up (mo)	Outcome	Postoperative nocturnal pain
Gudas^[29]^	1	2022	M	50	Upper end of the radius	MRI	Arthroscopic resection of the shoulder	12	Ranges of motion	No
Alrassasi^[25]^	1	2021	F	28	Elbow joint	radiography, CT, MRI	Arthroscopic resection of the elbow	12	Ranges of motion	No
Park^[20]^	1	2021	F	13	Posterior tibial area	radiography, CT	Arthroscopic knee resection	3	Lysholm, WOMAC	No
Plecko^[8]^	4	2021	3M/1F	23.2	Intra-articular area of the knee	CT, MRI, bone scan	Arthroscopic knee resection	24	Ranges of motion	No
Tsukada^[30]^	1	2020	M	17	Juxta-articular area of the calcaneus	radiography, CT, MRI	Subtalar arthroscopy	24	Ranges of motion	No
Ozdemir^[31]^	1	2020	M	17	Talar neck	CT, MRI	Arthroscopic resection of ankle	12	Ranges of motion	No
Spiker^[27]^	40	2018	24M/15F	24.5	Hip	CT, MRI	Arthroscopic hip resection	27	mHHS, iHOT33, HOS-ADL,	No

CT = computed tomography, HOS-ADL = Hip Outcome Score-Activity of Daily Living, iHOT = International Hip Outcome Tool, mHHS = modified Harris score, MRI = magnetic resonance imaging, OO = osteoid osteoma, VAS = visual analogue scale.

## Author contributions

**Conceptualization:** Yinhao He, Zhi-Xing Tu, Hui Zeng, Qiang Peng, Tie-Zhu Chen.

**Data curation:** Yinhao He, Hong-Wen Chen, Hui Zeng, Tie-Zhu Chen.

**Formal analysis:** Yinhao He, Xiaosheng Li, Zhi-Xing Tu, Hong-Wen Chen, Tie-Zhu Chen.

**Investigation:** Tie-Zhu Chen.

**Methodology:** Yinhao He, Tie-Zhu Chen.

**Project administration:** Xiaosheng Li, Tie-Zhu Chen.

**Resources:** Tie-Zhu Chen.

**Software:** Tie-Zhu Chen.

**Supervision:** Xiaosheng Li, Tie-Zhu Chen.

**Validation:** Xiaosheng Li, Zhi-Xing Tu, Tie-Zhu Chen.

**Visualization:** Yinhao He, Tie-Zhu Chen.

**Writing – original draft:** Yinhao He.

**Writing – review & editing:** Tie-Zhu Chen.
